# Reproducibility and Repeatability in Focus: Evaluating LVEF Measurements with 3D Echocardiography by Medical Technologists

**DOI:** 10.3390/diagnostics14161729

**Published:** 2024-08-09

**Authors:** Marc Østergaard Nielsen, Arlinda Ljoki, Bo Zerahn, Lars Thorbjørn Jensen, Bent Kristensen

**Affiliations:** Department of Nuclear Medicine, Herlev University Hospital, 2730 Herlev, Denmark; arljinda.ljoki@regionh.dk (A.L.); bo.zerahn@regionh.dk (B.Z.); lars.thorbjoern.jensen@regionh.dk (L.T.J.); bent.kristensen.01@regionh.dk (B.K.)

**Keywords:** 3D echocardiography, left ventricular ejection fraction (LVEF), reproducibility, repeatability

## Abstract

Three-dimensional echocardiography (3DE) is currently the preferred method for monitoring left ventricular ejection fraction (LVEF) in cancer patients receiving potentially cardiotoxic anti-neoplastic therapy. In Denmark, however, the traditional standard for LVEF monitoring has been rooted in nuclear medicine departments utilizing equilibrium radionuclide angiography (ERNA). Although ERNA remains a principal modality, there is an emerging trend towards the adoption of echocardiography for this purpose. Given this context, assessing the reproducibility of 3DE among non-specialized medical personnel is crucial for its clinical adoption in such departments. To assess the feasibility of 3DE for LVEF measurements by technologists, we evaluated the repeatability and reproducibility of two moderately experienced technologists. They performed 3DE on 12 volunteers over two sessions, with a collaborative review of the results from the first session before the second session. Two-way intraclass correlation values increased from 0.03 to 0.77 across the sessions. This increase in agreement was mainly due to the recognition of false low measurements. Our findings underscore the importance of incorporating reproducibility exercises in the context of 3DE, especially when operated by technologists. Additionally, routine control of the acquisitions by physicians is deemed necessary. Ensuring these hurdles are adequately managed enables the adoption of 3DE for LVEF measurements by technologists.

## 1. Introduction

Left ventricular ejection fraction (LVEF) is the main variable of choice for monitoring heart function in cancer patients receiving potentially cardiotoxic treatments [1]. Precise evaluation of LVEF is therefore important since it guides therapeutic decision making and has prognostic implications [2]. While three-dimensional transthoracic echocardiography (3DE) is the recommended first-line modality for LVEF monitoring, its effectiveness hinges not only on the operator’s skill but also on the agreement between observers. This highlights a critical aspect: the need to assess and ensure reproducibility of 3DE measurements, particularly when operated by technologists or other non-specialized medical personnel. Inconsistent results across different operators could significantly influence clinical decision making and subsequent outcomes. Therefore, our observational study primarily focused on evaluating the reproducibility and repeatability of real-time 3DE (RT-3DE) in the hands of technologists over a two-month period. The null hypothesis tested was whether the reproducibility of RT-3DE remains unchained, irrespective of the operator, within this timeframe.

## 2. Background

Two-dimensional echocardiography (2DE) dominates global practice for monitoring cancer-therapy-related cardiac dysfunction (CTRCD) [3]. Definitions and severity vary, but generally, non-symptomatic CTRCD can be defined as a significant decrease in LVEF (≥10 percentage points) to below the lower limit of normal (50–53%) [1,4].

Recent European guidelines favor 3DE or cardiac MR (CMR) for serial LVEF estimation [5]. 3DE, whether real-time or not, offers superior accuracy and lower observer variability when compared to 2DE [6,7,8]; however, 3DE is more dependent on good acoustic windows and patient cooperation. Meanwhile CMR, the gold standard for volumetric assessment, is less suitable for serial monitoring for CTRCD given its limitations of cost and availability. 

The shift towards 3DE and CMR has relegated the once staple equilibrium radionuclide angiocardiography (ERNA) to a secondary role in LVEF estimation, primarily due to concerns of radiation exposure from serial scans [9,10,11]. However, the guidelines base this recommendation on the tracer doses utilized for planar ERNA, an older technique that employs significantly higher radiotracer doses compared to what is possible utilizing cadmium–zinc–telluride (CZT)-based detectors [12]. The advantage of ERNA in general is its high accuracy and precision when compared with CMR [13], and its already impressive reproducibility can be further enhanced with CZT-based detectors [14]. It is also worth noting that other nuclear modalities such as myocardial perfusion imaging (MPI) including Rb-82 positron emission tomography (Rb-PET) and ^99m^Tc-Sestamibi-SPECT also are fully capable of providing highly reproducible LVEF assessments [15,16].

These nuclear modalities all have in common that they are less user-dependent than 2DE [17]; however, it remains to be demonstrated if non-specialized health care professionals such as technologists can wield 3DE to reliably detect changes within ±10 percentage points. 

Studies investigating the reproducibility of 3DE among non-specialized medical staff are less common in real clinical settings. Nonetheless, previous research has provided valuable insights into this area. Notably Guppy-Coles et al. demonstrated the feasibility of training cardiac nurses in the analysis of pre-acquired 3D echocardiographic data [18]. Building upon this foundation, their subsequent research further revealed the potential for cardiac nurses to not only analyze but also acquire 3DE images for LVEF assessment. The results demonstrated a reasonable degree of agreement between the cardiac nurses and experienced echo-sonographers. However, in both studies, reproducibility was determined by analyzing the same image twice rather than through repeated acquisitions, and the Bland–Altman limits of agreement (LOA) for the nurses versus the echo-sonographers exceeded 10 percentage points without accompanying confidence intervals [19]. 

Our observational study was designed to evaluate the reproducibility of LVEF measurements made by technologists with intermediate experience. The objective was to evaluate its potential as a complete or partial substitute for the currently employed CZT-ERNA technology.

## 3. Materials and Methods

This longitudinal prospective observational study, conducted at the Department of Nuclear Medicine, Herlev University Hospital, Denmark, focused on evaluating the repeatability, reliability, and reproducibility of LVEF measurements from RT-3DE by two technologists. The participant cohort included twelve hospital staff volunteers and consisted of six age-matched men and women, all without known heart disease.

After receiving training from the system’s vendor, the technologists applied RT-3DE for LVEF estimation in patients over an eight-month period, amounting to roughly 250 days of experience. During this period, the first technologist (OP1) successfully acquired usable 3D images in 75% (661/881) of the sessions, while the second technologist (OP2) had a success rate of 63% (349/558).

This study was conducted where each technologist performed three replicate LVEF measurements on each participant during two separate sessions, denoted as M1 and M2. To minimize a possible “carry-over” learning effect, the sessions were performed 1–2 months apart.

Between each replicate, the transducer was completely removed from the chest, while the participant remained in the left lateral decubitus position. After the scan, the technologists provided a combined grading of image quality and practical difficulty. The grading scale ranged from 0 to 3, where 0 indicated no difficulty, 1 a somewhat challenging acquisition, 2 a very difficult acquisition, and 3 an impossible task.

Operators were required to provide justification for scores of 1, 2, or 3, citing specific challenges such as poor endocardial definition or subjected-related issues like suboptimal positioning or problems with breath-hold maneuvers.

### 3.1. Real-Time Three-Dimensional Transthoracic Echocardiogram

Real-time full-volume three-dimensional transthoracic echocardiography was conducted using the Acuson SC2000 (Siemens Ultrasound, Mountain View, CA, USA) cardiovascular ultrasound system, equipped with a 4Z1c-phased array real-time volume transducer operating at a frequency range of 1.5–3.5 MHz.

Complete left-ventricular volumes were captured from the apical view during every cardiac cycle over a period of three consecutive cycles. Patients were positioned in the left lateral decubitus orientation and instructed to perform breath-hold maneuvers by the operator.

Subsequent analysis of the full-volume images was carried out off-line utilizing eSie LVA version 5.1. This software features a fully automated, knowledge-based algorithm for endocardial detection, removing the need for manual delineation of endocardial borders by the operators [20].

### 3.2. Statistical Analysis

The statistical analyses were conducted using the following:

‘R’ version 4.2.2 (R Core Team (2022): A language and environment for statistical computing, R Foundation for Statistical Computing, Vienna, Austria. URL https://www.R-project.org/ (accessed on 4 August 2024), including but not limited to the following packages: tidyverse (dplyr, ggplot2), LMMstar, irr, VCA, performance, and lme4.

**Figure A1:**
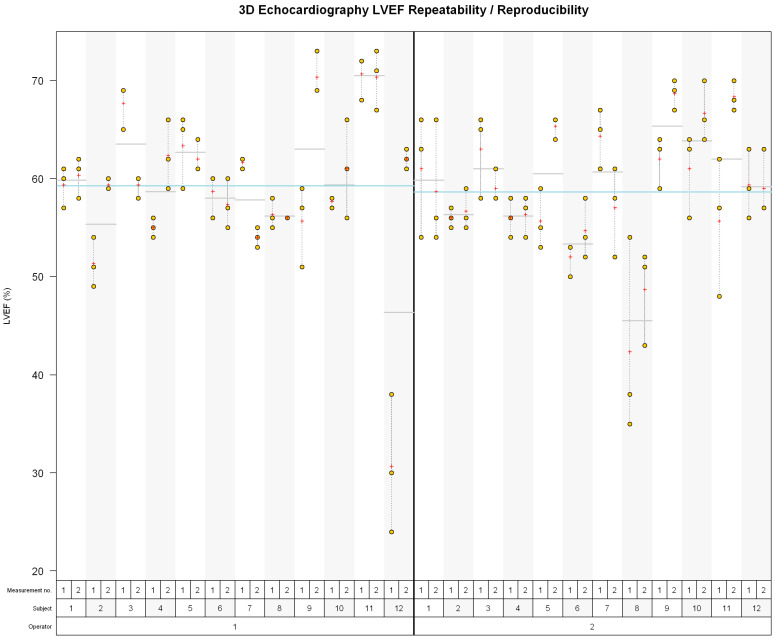
Variability chart of the total measurement dataset generated using the “VCR” R package. Individual measurements are depicted as points, with the red plus symbol representing the mean of each replicate set. Grey horizontal bars indicate the mean of all measurements for each subject, while light-blue bars represent the means of the entire measurement session.

**Figure A2:**
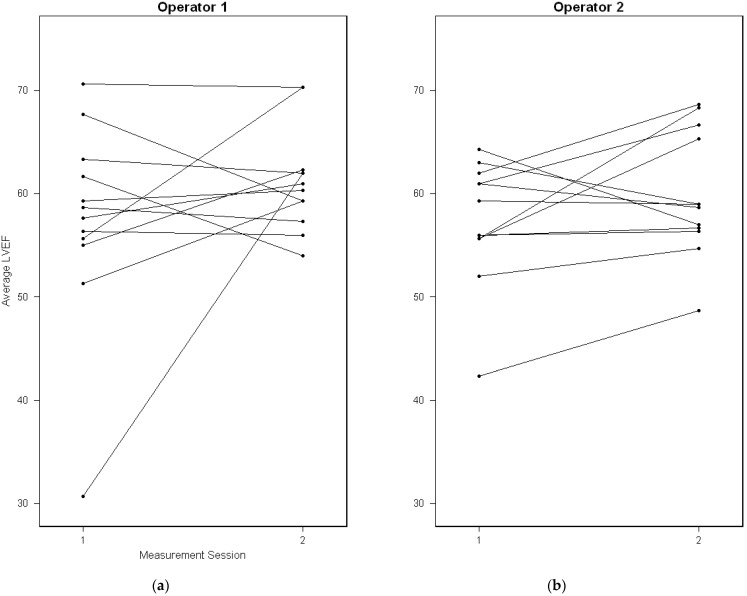
Paired-line plots or “spaghetti-plots”, representing the change in mean-LVEF between measurement sessions for operator 1 in panel (**a**) and operator 2 in panel (**b**).

Descriptive statistics included visualizations, medians, and ranges, and significance was determined using either Welch’s *t*-test or the Mann–Whitney U test, depending on the data distribution. The whole dataset can be viewed from Figure A1 in the Appendix A, and the trend between measurement sessions is visualized through Figure A2. 

Intraclass correlation coefficient (ICC) calculations were employed for evaluating operator agreement and consistency. We used a one-way model for intra-operator consistency and a two-way model for inter-operator agreement. For the Bland–Altman plot, the confidence intervals for the LOA were calculated using exact methods, providing a more precise estimation of the agreement levels [21]. 

It is worth noting ICC’s limitations: Its values can vary based on the chosen model, type, and measures; it is sensitive to subject variability, potentially affecting reliability assessments; and its statistical nature can complicate clinical interpretation [22]. Appendix A contains Table A1, which serves as a guide for interpreting ICC values. 

A linear mixed model (LMM) was constructed where operators were treated as a fixed effect to assess if they exerted a systematic, significant influence on the LVEF measurements. To further explore the operators’ impact on the variability of the measurements, we developed a second model where the operators were considered a random effect. We made this adjustment to determine if the operators’ influence varied across the subjects’ measurements. In both models, variance component analysis included random effects for subjects, measurement sessions, and replicates, allowing us to quantify the variance attributed to each factor. These variances were used to calculate the repeatability and reproducibility coefficients detailed in Appendix A.

An important part of the statistical analysis is assessment of the assumptions and quality of the model. These analyses were carried out with the R package performance.

## 4. Results

Six males and six females were studied. Descriptive variables are presented in Table 1. Significant differences in all volumetric variables (LVEF, EDV, and ESV) were observed between men and women, as evidenced by the Mann–Whitney U test (*p* < 0.01).

**Figure 1:**
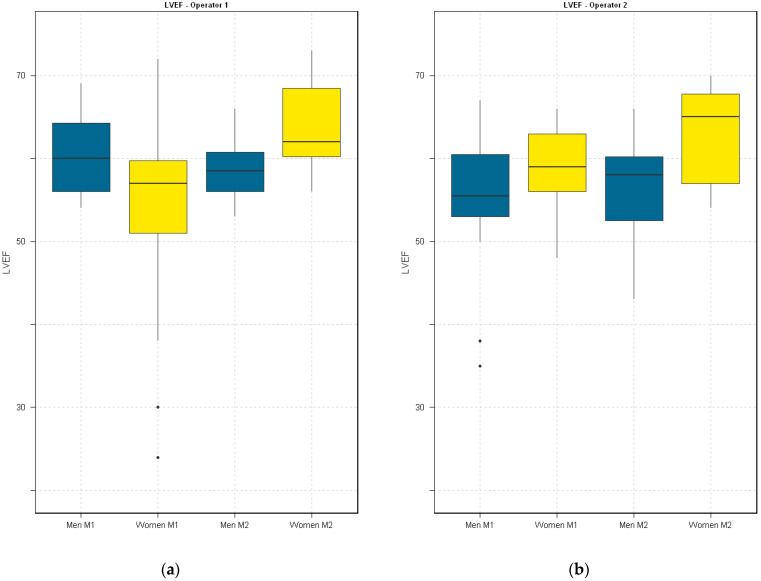
Boxplots of LVEF measurements by OP1 (**a**) and OP2 (**b**), further split by sex and measurement session.

The boxplots show small differences in LVEF measurements between operators. Both operators observed an increase in LVEF values for women from M1 to M2 (Figure 1). No significant differences were found when comparing M1 to M2 for each gender for each operator using both the paired *t*-test and the Wilcoxon signed-rank test.

**Figure 2:**
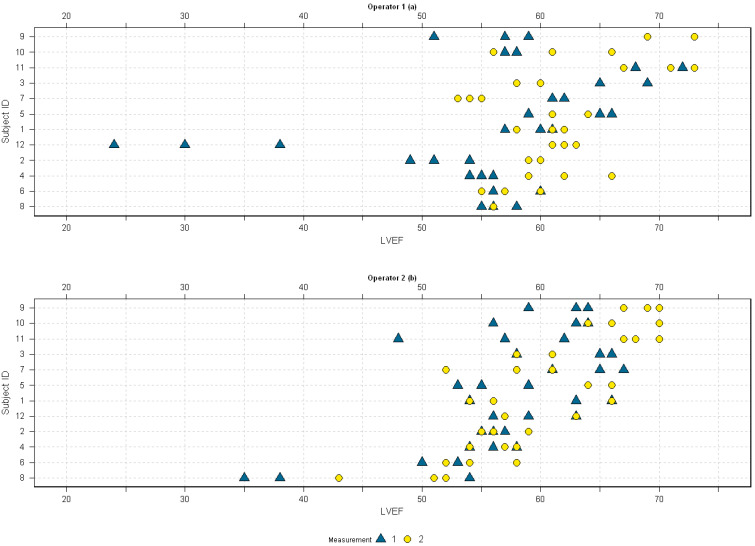
Strip-dot plots of LVEF percentages by OP1 (**a**) and OP2 (**b**). Triangles represent M1 and circles M2.

Inspection of the strip plots (Figure 2) identified seven deviant measurements under 50% LVEF, specifically (a) OP1’s measurements for subject 12 in M1 (replicates 1, 2, and 3); (b) OP2′s measurements for subject 8 (two outliers in M1 and one in M2) and one outlier from subject 11 in M1.

**Figure 3:**
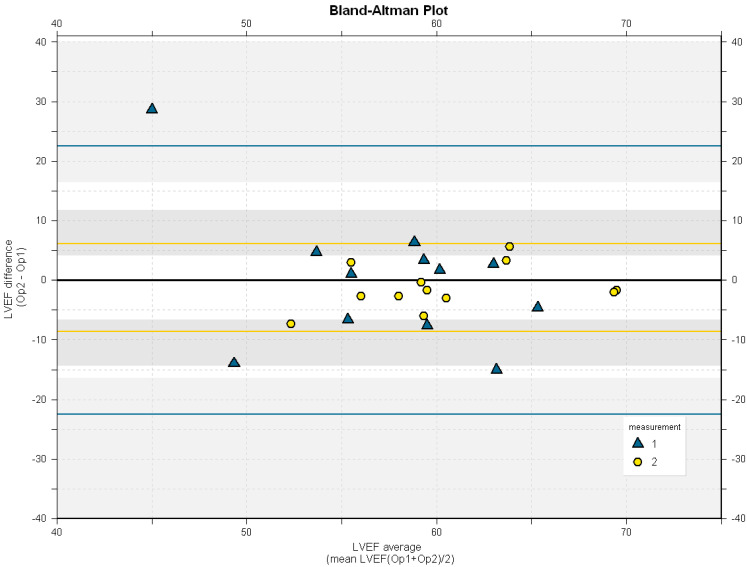
Bland–Altman plot with LOA (solid, colored lines) with confidence intervals (shaded grey areas). Triangles represent M1 and circles M2. The x-axis represents the average LVEF of OP1 and OP2, while the y-axis represents the difference.

The Bland–Altman plot (Figure 3) illustrates the agreement of LVEF between OP1 and OP2 across the measurement sessions. The LOA for M1 spanned 45 percentage points (22.5 to −22.5), while LOA for M2 spanned 14.7 percentage points (6.1 to −8.6). 

Removing the outlier (top left corner) narrowed the LOA of M1 to 29.3 percentage points (12.1 to −17.2). 

Table 2 shows one-way ICC calculations, indicating the consistency of the operators in M1 and M2. The consistency of OP1 decreased from 0.91 in M1 to 0.80 in M2. In contrast, OP2′s consistency increased from 0.55 in M1 to 0.75 in M2. All one-way ICC calculations were statistically significant (*p* < 0.001).

Table 3 details two-way ICC calculations for assessing observer agreement. The ICC for M1 was near 0, increasing to 0.35 after outlier removal, yet remained non-significant. The ICC for M2 was 0.77, improving slightly to 0.79 after excluding outliers; both these values were statistically significant.

The two LMMs detailed in the statistical section where operators were considered either a fixed or random effect differed slightly. When considered as a fixed effect, operators were not statistically significant (*p* = 0.277). The estimated average decrease in LVEF was 1 percentage point for operator 2 compared to operator 1. Treating operators as a random effect accounted for only 2.5% of the overall variance. The estimated intercept for LVEF was 60.5. The second model’s results, which considered operator as a random effect, are summarized below in Table 4.

Despite the relatively modest sample size, model checking did not reveal serious deviations from the model’s assumptions.

Using these standard deviations, we calculated the repeatability coefficient for within-subject variation as 7.1. To evaluate the reproducibility of LVEF measurements, we applied the same formula but used different variance parameters. With variations between sessions and different operators, the reproducibility coefficient was determined to be 10.3. When we isolated the variability between sessions alone, the coefficient decreased to 7.9.

In the evaluation of scan performance, there were no patterns discernible when both operators were considered together. However, OP1 used the performance rating “somewhat difficult” (rating 2) 50% less, instead choosing “no problem” (rating 1) or “very difficult” (rating 3) from M1 to M2.

## 5. Discussion

We assessed the repeatability, reproducibility, agreement, and consistency of RT-3DE LVEF estimations by intermediately experienced technologists on 12 healthy subjects. 

Our results show a marked increase in agreement between the two observers across measurement sessions. Additionally, our LMM analysis and coefficient calculations estimate the variability expected in LVEF values from replicate measures, between sessions, and between operators. With these insights, we are confidently positioned to identify clinically relevant changes in LVEF of ±10 percentage points, providing a level of precision that supports informed clinical decision making.

Following outlier exclusion, as detailed above Figure 2, LVEF values aligned with established normal ranges for a Scandinavian population [23]. End-diastolic and end-systolic values also aligned with publications utilizing the same echocardiographic system [24]. Gender-specific differences in volumetric measurements were anticipated, as this has been documented across various imaging modalities [25,26]. The study design ensured no significant age variance between groups. 

Differences between operators were especially pronounced during M1, which translated to minimal agreement, evident by a low ICC (Table 3) and wide LOAs (Figure 3). Removing outliers improved this agreement, but it remained poor. The measurements in M2 demonstrated significantly improved agreement, which was marginally improved after excluding outliers. We attribute this increase in agreement primarily to the discussions and reflections that followed M1. These included a deeper focus on recognizing the necessary image quality for accurate endocardial tracing.

The Bland–Altman plot (Figure 3) illustrates the LOA between the two operators’ LVEF measurements across M1 and M2. The LOA for M2 indicates that changes in LVEF exceeding 10 percentage points (−10–10) are discernible (in approximately 95% of cases) when different operators conduct the measurements. However, to enhance measurement precision, our future aim is to narrow the outer bounds of the LOAs CIs to within ±10 percent. 

The outliers identified on the strip plots (Figure 2) were the main driver of the disparities observed between the boxplots (Figure 1). Reviewing the images confirmed suboptimal endocardial tracing by the automatic algorithm due to poor endocardial definition. After exclusion of these, the boxplots between the observers were nearly identical. 

The small increase in LVEF for female participants from M1 to M2 was consistent for both operators even after outlier exclusion. Combined with a small increment in heart rate, this suggests that the increase in LVEF is most likely physiological in nature rather than the result of measurement error.

To ensure the inclusion of only physiologically accurate LVEF values in our linear mixed model, we excluded the outliers detailed in Figure 2. This was done to avoid bias and inaccuracies in representing variability. The repeatability coefficient of 7.1 indicates that replicate measurements on the same subject by the same operator are expected to fall within ±7.1 percentage points in 95% of the time, providing an estimate of measurement error. This information is vital for physicians, as it offers a margin of error to consider when interpreting individual measurement results. 

For reproducibility, we calculated the coefficient from the variation contributed by operators and between sessions. The resulting value of 10.3 suggests that an LVEF change greater than 10 percentage points is likely significant in practice. The variation between sessions without the effect of the operators was estimated at 7.9 percentage points. This lower value indicates the extent of LVEF variability over time within our healthy study population, highlighting another critical factor for physicians evaluating measurements longitudinally.

Our analysis revealed no significant systematic differences between operators. When modeled as a fixed effect, differences among operators were statistically insignificant; when treated as a random effect, operator-related variance contributed minimally to the total variance. This indicates that operator influence is predominantly random, aligning more with measurement error associated with the replicate measurement process. About 27% of the variance remains unexplained by our model, potentially encompassing factors such as environmental variations, subtle physiological changes not captured by other variables, or other unmeasured aspects of the LVEF measurement process.

To enhance the reliability of LVEF measurements in actual patient cohorts, we recommend conducting additional repeated measures, particularly for LVEF values around 50%. Furthermore, involving the reading physician in selecting the most representative measurement for reporting could improve accuracy and relevance.

Our evaluation of intra-operator consistency is summarized in Table 2. We observed a high consistency for OP1 overall, which decreased slightly in M2. However, despite high consistency, this operator performed false low measurements in subject 12 during M1, and this highlights that precision does not inherently imply accuracy. The discovery and subsequent discussion of these false low measurements could have affected the confidence of OP1, perhaps explaining the small decrease in ICC. Conversely, the ICC of OP2 improved from 0.55 to 0.75 across series, indicating potential learning-curve effects and increased confidence with the measurement protocol.

Interpreting our results in terms of completion rates might be viewed as somewhat forced. However, given the context of using RT-3DE for LVEF monitoring, we find that the measurements for subject 12 by OP1 are unreliable. The baseline measurements were consistently too low, making the subsequent M2 replicates clinically irrelevant, as they do not allow for accurate tracking of changes over time. Meanwhile, the outlier results from OP2, when grouped with measurements that displayed more realistic contouring, provide an opportunity for physicians to select the most clinically relevant replicate.

By excluding subject 12’s results, OP1 achieved a completion rate of 92%. OP2, after discarding the four problematic replicates, reached a completion rate of 94%. These rates, though impressive, may not accurately reflect the broader patient demographic referred to the department. These patients are characterized by older age and higher body mass index—factors known to affect the quality of the acoustic window [27].

The issue of operator skill and its influence on reproducibility, particularly among non-specialized medical personnel in the context of RT-3DE, remains largely unexplored. This study highlights the variability in ICC values between operators, potentially influenced by several factors. OP1, who had more experience, demonstrated better consistency when compared to OP2.

Furthermore, the difference in experience, as detailed in the methods section, as well as the impact of the coronavirus pandemic, which necessitated a nearly two-month pause in scanning activities during a nationwide lockdown in Denmark in 2020, could have exacerbated these initial discrepancies. This interruption may have contributed to a drift in agreement between the operators, underlining the challenges faced in maintaining consistency in clinical measurements during unprecedented disruptions.

It remains unclear whether the operators were initially in closer agreement at the start of their patient scans before this study and subsequently diverged over time. However, this aspect is of lesser importance. The crucial observation is their eventual convergence towards greater agreement and enhanced ICC values as the study progressed. This trend suggests that discrepancies in operator skill can be markedly reduced with collaborative review of 3DE acquisitions.

The limitations of this pilot observational study include its small sample size and LVEF range above 50%. Secondarily, we are unable to verify the accuracy of the measurements obtained. This could have been remedied by including a third “expert” operator or by directly comparing the measurements to cardiac magnetic resonance; the latter, however, would significantly increase the cost and complexity of the study. Moreover, the rather modest sample size, all else being equal, entails a higher risk of false-positive results.

Additionally, OP1′s consistent measurement error across all replicates for subject 12 during the first measurement session underscores the necessity of alternative methods in cases of poor echocardiogram quality. Our current modality for LVEF determination, CZT-ERNA, boasts a high feasibility rate exceeding 98% in our patient cohort, with Rb-PET expected to achieve even higher success rates. These modalities could provide robust alternatives for LVEF quantification.

Furthermore, we did not evaluate the potential influence of chest wall conformation on the repeatability of our measurements. Previous research has shown that chest wall shape significantly affects the repeatability of 2D-derived LVEF measurements [28]. Future studies could consider these factors to better understand their impact on echocardiographic measurements.

## 6. Clinical Implications

RT-3DE proves effective for LVEF assessments by non-specialized medical personnel, provided that specific controls are in place to ensure reliability. Key practices include reproducibility exercises to manage inter-operator variability and improve agreement as well as physician oversight to address potential inaccuracies from suboptimal endocardial contouring due to poor image quality.

In individuals without cardiovascular diseases, natural LVEF variability over time was estimated at 8 percentage points. The within-session measurement error was estimated at 7 percentage points. No systematic variability due to operators was detected.

## 7. Conclusions

With our study, we demonstrated a significant improvement in observer agreement between two technologists using RT-3DE. This enhanced agreement is fundamental for identifying clinically relevant changes in LVEF of ±10 percentage points.

The improvement was the result of a collaborative review of measurements from the first measurement session. Such “reproducibility exercises” are deemed essential, particularly with major changes in personnel or technology and at regular intervals. Additionally, when measurements are made by non-specialized medical personnel, such as our moderately experienced technologists, physician oversight of image quality is crucial.

Although our sample size was small and consisted of healthy volunteers with normal LVEF values, our results support the use of RT-3DE by non-specialized medical personnel under the specified conditions.

Moving forward, we aim to study observer agreement and the precision of LVEF measurements in actual patient cohorts using RT-3DE. We plan to incorporate a secondary method like CZT-ERNA when echocardiogram quality is suboptimal. Exploring other methods, such as Rb-PET, will also be a key research focus.

## Figures and Tables

**Table 1:** Descriptive variables presented as medians and (ranges).

Variable	Men	Women
Age	49 (28–66)	47 (26–62)
Body mass index (BMI)	23 (21–31)	22 (20–32)
Heart rate	58 (45–80)	55 (46–80)
LVEF	58 (35–69)	60 (24–73)
End-diastolic volume	124 (97–193)	117 (58–184)
End-systolic volume	53 (32–92)	43 (19–84)
LVEF (outliers excluded)	58 (50–69)	61 (49–73)

**Table 2:** One-way ICC calculations of LVEF replicates grouped by operator and measurement session.

Operator, Measurement Session	ICC * (95% Confidence Intervals)
OP1, M1	0.91 (0.79–0.97) ^1^
OP1, M2	0.80 (0.57–0.93) ^1^
OP2, M1	0.55 (0.21–0.82) ^1^
OP2, M2	0.75 (0.49–0.91) ^1^

^1^ *p* < 0.001; * intraclass correlation coefficient.

**Table 3:** Two-way ICC calculations of agreement between operators using LVEF means of the replicates grouped by measurement series.

Measurement Session	ICC (95% Confidence Intervals)
M1	0.03 (−0.61–0.60)
M2	0.77 (0.39–0.93) ^1^
M1 *	0.35 (−0.31–0.77)
M2 *	0.79 (0.45–0.94) ^1^

* After exclusion of outliers; ^1^ *p* < 0.001.

**Table 4:** LMM variance component analysis with operators treated a random effect.

Variance Component	Standard Deviation (%,Total)
Within-subject (replicates)	2.6 (21%)
Between-subject	2.7 (24%)
Between-measurement	2.8 (26%)
Inter-observer	0.9 (2%)

## Data Availability

Dataset available upon request from authors.

## Appendix A

Regarding sample size for such a pilot study, “the recommendation of a sample size of 12 per group” was followed, viz. 6 women and 6 men covering the same age range. The justifications for this sample size are based on rationale about feasibility and precision about the mean and variance [29].

The analyses for repeatability, reproducibility, and reliability were carried out according to the recommendations described in detail in the following papers and books [30,31,32,33,34,35].

### Appendix A.1. Unlinked Data

The repeated measurements on a subject are unlinked (or unpaired) if the measurements from the two methods/measurement sessions are obtained separately, and a measurement session’s multiple measurements on a subject are independent replications of the same underlying measurement. A replication is a repeated a measurement under identical conditions, ensuring that the true value of the subject remains unchanged during the measurement period. The data for the present analysis are assumed to be unlinked.

### Appendix A.2. Repeatability

Repeatability of measurements refers to the variation in repeat measurements made on the same subject under identical conditions. This means that measurements are made by the same instrument or method and by the same observer if human input is required, and the measurements are made over a short period of time, over which the underlying value can be considered to be constant. Variability in measurements made on the same subject in a repeatability study can then be ascribed only to errors due to the measurement process itself.

The repeatability is also called the smallest real difference, minimal detectable change, and minimum clinical difference because it is the smallest difference that can be interpreted as evidence for a real change in the subject’s true value.

In a repeatability study, the possibility of bias between measurements is excluded so that agreement between measurements made on the same subject depends only on the within-subject standard deviation (*SD*), which measures the size of measurements errors. One way to describe the agreement is to report an estimate of the within-subject *SD*, which is the same as the *SD* of the measurement errors. An alternative is to report the *SD* of the differences between two measurements made on the same subject. This is equal to the following:

$$\sqrt{2}\cdot {SD}_{within-subject}$$

A further alternative is to report the estimated repeatability coefficient (*RC*) [36], which is defined by the following:

$$RC=1.96\cdot \sqrt{2}\cdot {SD}_{within-subject}$$

If the differences between two measurements made on a subject are approximately normally distributed, then in the long term, one can expect the absolute difference between two measurements on a subject to differ by no more than the repeatability coefficient on 95% of occasions—unless a pronounced (significant) change has occurred, e.g., due to a cardiotoxic treatment.

### Appendix A.3. Reproducibility

Reproducibility refers to the variation in measurements made on a subject under changing conditions. The changing conditions may be due to different measurement methods or instruments being used, measurements being made by different observers, or measurements being made over time, within which the “error-free” level of the variable could undergo non-negligible change.

### Appendix A.4. Reliability

The reliability of a measurement method is defined as the proportion of variation in observed measurements that is not explained by the error variation inherent in the method. Reliability relates the magnitude of the measurement error in observed measurements to the inherent variability in the “error-free”, “true”, or underlying level of the quantity between subjects.

These measures of variability is often expressed as *SD*s, and formally, reliability is defined as follows:

$$Reliability=\frac{{\left({SD}_{subjects^{\prime }\;true\;value}\right)}^{2}}{{{\left({SD}_{subjects\;true\;values}\right)}^{2}+({SD}_{measurement\;error})}^{2}}$$

If the reliability is high, measurement errors are small in comparison to the true differences between subjects so that subjects can be relatively well distinguished (in terms of the quantity being measured) based upon of the error-prone measurements.

Conversely, if measurement errors tend to be large compared with the true differences between subjects, reliability will be low because differences between the measurements of two subjects could be due purely to error rather than to a genuine difference in their true values.

The reliability parameter is also known as an intraclass correlation coefficient (ICC), as it equals the correlation between any two measurements made on the same subject. Reliability takes values between zero and one, with a value of one corresponding to zero measurement error and a value of zero meaning that all the variability in measurements is due to measurement error. As a dimensionless quantity, it may be difficult to interpret, and deciding what value constitutes sufficiently high reliability is often carried out in a subjective fashion. The following table may serve as a guide for interpreting the ICC:

**Table A1:** Interpreting ICC values.

Reliability Outcome	Koo et al. [37]	Cicchetti et al. [38]
Poor	<0.50	<0.40
Fair	0.50–0.75	0.40–0.60
Good	0.75–0.90	0.60–0.75
Excellent	0.90–1.00	0.75–1.00

The reliability of a method is a measure of its relative precision. A high value of reliability indicates that the error variation is small compared to the variation in the error free values.

The expression for reliability shows that it depends on the error variation of the method as well as the heterogeneity (or the between subject variation) in the population. In particular, the reliability increases as the population heterogeneity increases even if the precision of the method does not change. Thus, care must be taken in interpreting reliability, especially comparison with ICCs in other (similar) studies. Reporting the between individual and within individual variation together with the ICC is therefore important.
